# Effects of non-invasive vagus nerve stimulation on clinical symptoms and molecular biomarkers in Parkinson’s disease

**DOI:** 10.3389/fnagi.2023.1331575

**Published:** 2024-02-07

**Authors:** Banashree Mondal, Supriyo Choudhury, Rebecca Banerjee, Akash Roy, Koustav Chatterjee, Purba Basu, Ravi Singh, Saptak Halder, Shantanu Shubham, Stuart N. Baker, Mark R. Baker, Hrishikesh Kumar

**Affiliations:** ^1^Institute of Neurosciences Kolkata, Kolkata, India; ^2^Faculty of Medical Sciences, Newcastle University, Newcastle upon Tyne, United Kingdom; ^3^Department of Neurology, Royal Victoria Infirmary, Newcastle upon Tyne, United Kingdom; ^4^Department of Clinical Neurophysiology, Royal Victoria Infirmary, Newcastle upon Tyne, United Kingdom

**Keywords:** vagus nerve stimulation, Parkinson’s disease, gait, neuroinflammation, oxidative stress

## Abstract

Non-invasive vagus nerve stimulation (nVNS) is an established neurostimulation therapy used in the treatment of epilepsy, migraine and cluster headache. In this randomized, double-blind, sham-controlled trial we explored the role of nVNS in the treatment of gait and other motor symptoms in Parkinson’s disease (PD) patients. In a subgroup of patients, we measured selected neurotrophins, inflammatory markers and markers of oxidative stress in serum. Thirty-three PD patients with freezing of gait (FOG) were randomized to either active nVNS or sham nVNS. After baseline assessments, patients were instructed to deliver six 2  min stimulations (12  min/day) of the active nVNS/sham nVNS device for 1  month at home. Patients were then re-assessed. After a one-month washout period, they were allocated to the alternate treatment arm and the same process was followed. Significant improvements in key gait parameters (speed, stance time and step length) were observed with active nVNS. While serum tumor necrosis factor- α decreased, glutathione and brain-derived neurotrophic factor levels increased significantly (*p* < 0.05) after active nVNS treatment. Here we present the first evidence of the efficacy and safety of nVNS in the treatment of gait in PD patients, and propose that nVNS can be used as an adjunctive therapy in the management of PD patients, especially those suffering from FOG.

**Clinical trial registration**: identifier ISRCTN14797144.

## Introduction

For more than 20 years, surgically implanted vagus nerve stimulation (VNS) has been recognized as an adjuvant neuromodulation therapy for epilepsy (Terry, 2009). Additionally, it has proven effective in treating depression, cluster headache, and migraine (Mauskop, 2005). The *nucleus tractus solitarius* and *locus coeruleus* are believed to be the primary targets of VNS, although the precise mechanisms are still mostly unknown (Kraus et al., 2007; Oshinsky et al., 2014). Handheld non-invasive VNS (nVNS) devices have recently been developed, simplifying this technique of treatment (Yuan and Silberstein, 2016). The capacity to test the intervention in a variety of medical conditions without running the risk of surgical or post-operative complications (Ben-Menachem et al., 2015) is only one benefit of this strategy. According to several studies, VNS may have anti-inflammatory effects in addition to its impact on central neural networks (Corcoran et al., 2005; Majoie et al., 2011). As a result, possible uses have been suggested for a variety of inflammatory diseases, such as rheumatoid arthritis, sepsis, diabetes, and cardiovascular conditions (Bonaz et al., 2016). It is interesting to note that neuroinflammation has been connected to the pathophysiology of Parkinson’s disease (PD) and several other neurodegenerative diseases (Akiyama et al., 2000).

The most widespread and second most common neurodegenerative movement disorder, PD is characterized by bradykinesia, resting tremor, rigidity, and postural instability (Sveinbjornsdottir, 2016), which are the syndrome defining clinical features; however, other phenotypic subtypes (and phenotype-genotype associations) are recognized (Dulski et al., 2022). Patients with PD struggle to walk at a normal pace and rhythm (Mondal et al., 2019a). When PD is at advanced stages, patients experience freezing of gait (FOG), feeling “glued to the ground” for seconds or minutes (Giladi et al., 2001). These symptoms are incapacitating and eventually worsen because of progressive degeneration within the nigrostriatal system (Riederer and Wuketich, 1976). Inflammation along with oxidative stress and altered cellular metabolism are undoubtedly the key participants in the pathophysiology of PD (Beal, 2003). Upregulation of neuroinflammatory mediators has been found in PD patients by our team (Chatterjee et al., 2020) and others (Wang et al., 2015). In order to slow the progression of the disease, inflammatory modulators have been thoroughly investigated (Klegeris et al., 2007); however, the results to date have been inconclusive.

It has recently been reported that VNS can improve mobility in a rat model of PD (Farrand et al., 2017) and two preclinical studies have shown that a single cervical nVNS application can improve gait in individuals with PD (Morris et al., 2019; Mondal et al., 2019a). There is mounting evidence that VNS can lower oxidative stress, regulate inflammatory cytokines, and strengthen anti-oxidative mechanisms (Chen et al., 2016). Whilst the anti-inflammatory effects of VNS could have important disease modifying actions in PD (Johnson and Wilson, 2018), these mechanisms are unlikely to account for the single dose effects of nVNS. Although the precise mechanisms by which VNS exerts its effects in PD remain largely unknown (Sun et al., 2013; Mondal et al., 2019b), the immediate improvements seen after a single application of nVNS in pilot studies are more likely to be the result of indirect activation of central neural circuitry, including noradrenergic projections from the *locus coeruleus* (Johnson and Wilson, 2018), a brain region implicated in the aetiopathogenesis of FOG (Ono et al., 2016). Despite the positive results of pilot studies of nVNS in PD, it is not apparent if or to what extent continuous stimulation might have long-lasting benefits (Hays et al., 2013; Lewine et al., 2019).

We investigated the effectiveness of cervical nVNS (gammaCore, ElectroCore, Inc., NJ, United States) as an addition to standard treatment for PD patients with FOG in a randomized double-blind sham-controlled cross-over trial. In order to evaluate the impact of chronic nVNS on neuroinflammation and neuroplasticity in PD patients, we also evaluated serum levels of specific indicators of inflammation and oxidative stress as well as brain derived neurotrophic factor (BDNF) in a subgroup of patients. Our results confirm that treatment with nVNS three times per day for 1 month improves gait and inflammatory biomarkers in blood in patients with PD.

## Methods

We recruited 33 PD patients of both sexes, aged 30–80, from the movement disorders outpatient clinic at a tertiary care hospital in Eastern India who had FOG. Only patients who were able to turn 180 degrees on the spot and walk continuously for at least 30 meters without assistance were included in the trial. Patients with baseline scores of 2 on both items 2.13 and 3.11 of the MDS- UPDRS rating scale, which are specific to FOG were included in the analysis. These patients were diagnosed in accordance with UK Brain Bank Criteria (Meara et al., 1999).

We excluded patients with i) early atypical parkinsonism (such as supranuclear gaze palsy), ii) vision impairment iii) concurrent local or systemic disorders (such as osteoarthritis or other neurological conditions) that could have an impact on gait, iv) deep brain stimulation surgery, v) implanted cardiac pacemaker, vi) metallic implants close to the stimulation site (such as fusion of cervical vertebrae), vii) uncontrolled hypertension, viii) recent myocardial infarction, or ix) known or suspected cardiovascular disease.

### Study methodology

**Figure 1:**
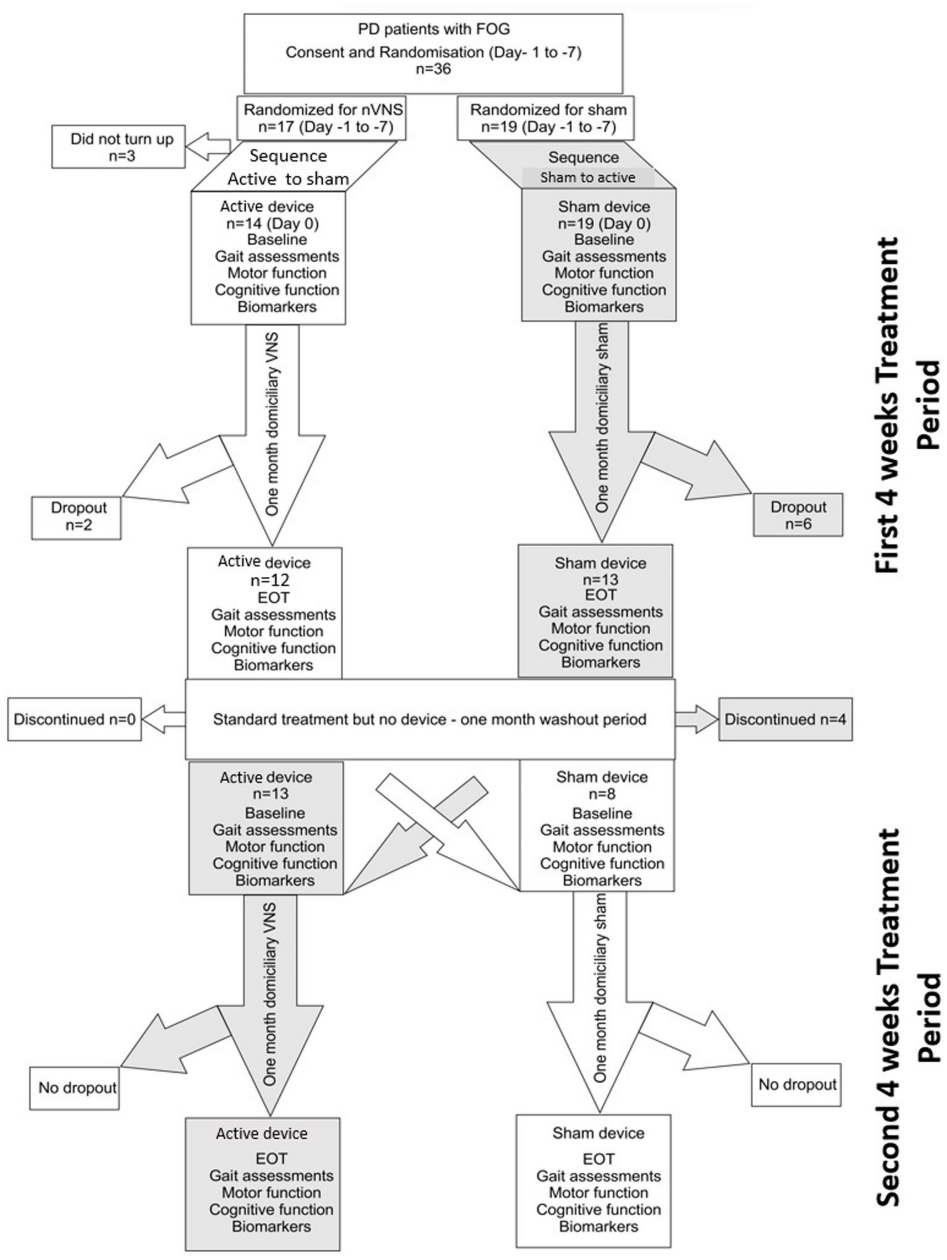
Consort diagram for the randomized cross over controlled trial comparing active non-invasive VNS (nVNS) and sham nVNS. PD, Parkinson’s disease; FOG, freezing of gait; n, number/sample size; VNS, Vagus nerve stimulation; EOT, End of treatment visit.

Each patient underwent four assessments during the 12-week study period (consort diagram; Figure 1). Prior to randomization, patients were evaluated for eligibility at the screening appointment based on a set of criteria, including a review of their medical history and current medications. Within 7 days following the consent process, patients were asked to come in for baseline evaluations before receiving their devices. This included an extensive neurological evaluation as part of a general physical examination. Clinical measures were used to evaluate the motor and non-motor symptoms of PD (see section below). On the same day, tests of cognition and gait were also conducted. Following an overnight L-dopa-free interval, all assessments were conducted in the OFF state. The patients were randomly assigned to either active nVNS or sham nVNS first (explained in the Treatment section). Patients and carers were instructed to apply the therapy at home for a month after receiving training on how to administer nVNS. After 4 weeks (the first treatment period), the patients came back for their follow-up appointment. Patients from the same cohort returned for a second follow-up appointment after a washout period of 4 weeks, when they were then assigned the alternative intervention for the second phase of the trial (second treatment period). At each of the four visits, the same set of evaluations were conducted.

A small number of patients only took part in the biomarker investigation. For the redox marker, serum samples from 14 patients in the active nVNS arm and 12 patients in the control arm were collected. Seven patients provided paired samples for the calculation of inflammatory biomarkers and for BDNF determination. Six subjects had their blood drawn twice (at the beginning and end of each treatment period). The samples from the remaining subjects, which were unpaired samples, only covered one treatment session.

### Randomization

Allocation of active and sham nVNS of devices was blinded and randomized in a 1:1 ratio. Simple randomization was done using a computer-generated list of random numbers (Random Allocation version 2.0). Active and sham nVNS devices could only be distinguished by their serial numbers. The commercial sponsor (electroCore, Inc.) sent the unblinded trial oversight committee (not involved in patient recruitment or evaluation) a comprehensive list of serial numbers and the stimulation mode of each device (sham or active) and its serial number. The distribution of devices was not disclosed to the researchers, site coordinators, or participants until the experiments were completed.

### Treatment

A proprietary frequency-modulated electrical stimulus (5 kHz sine wave stimuli of 1 ms duration at 25 Hz) was produced by the active nVNS device (electroCore, Inc.) at low voltage (24 V) and a maximum current output of 60 mA. The stimulation was applied to the neck near the vagus nerve using two stainless steel contact surfaces coated with conductive gel. The sham device (also provided by electroCore, Inc.) was identical in terms of appearance, weight, and user interface, and while it delivered detectable electrical stimulation to the skin (with a maximum output of 14 V and 24 mA), the sham stimulator’s proprietary low-frequency (0.1 Hz biphasic DC) delivery was specifically engineered not to activate the vagus nerve. Using the medial borders of the sternocleidomastoid muscle and the carotid pulse as anatomical landmarks, the treatment consisted of two, 2-min stimulation intervals delivered 5–10 min apart to the left vagus nerve to reduce any potential cardiac side effects (cardiac vagal efferents typically travel in the right vagus nerve). The intensity of the electrical stimulation was individualized based on the pain threshold of the patient. The maximum intensity was selected just below the pain threshold of the patient. For each participant, the identical stimulus intensity was applied throughout the entire investigation. We inquired about any adverse nVNS-related incidents. Every day, the intervention was given at three predetermined times: immediately after waking up, 6 to 8 h after the first treatment, and again 6 to 8 h after the second treatment.

### Assessments

At each visit, a set of clinical rating measures and gait analysis tools were used to evaluate PD-related motor and non-motor symptoms in each patient.

Gait analysis, the MDS-UPDRS scale (Pedersen et al., 2008), the freezing of gait questionnaire (Giladi et al., 2000), (FOG-Q) and the falls efficacy scale (Hauer et al., 2010) were used to evaluate motor function. Gait was evaluated using the Timed Up and Go test (Christopher et al., 2021) and an instrumented walkway (GaitRite, United States) (Webster et al., 2005). In addition to the questionnaire on freezing of gait (FOGQ), *post hoc* video gait evaluations were carried out to gauge the degree of FOG. The Mattis Dementia Rating Scale (Bezdicek et al., 2015) and the Mini Mental State Examination (Folstein et al., 1975) were two of the non-motor functional assessments. The rapid eye movement sleep behavior disorder (RBD) (Folstein et al., 1975; Stiasny-Kolster et al., 2007) screening questionnaire was one of the non-motor functional tests for cognition and sleep. In a smaller subset of individuals, serum biomarkers were assessed (see above). The Supplementary material contains a description of the assessment protocols in detail.

TNF-α, IL-6, IL-10, and BDNF were quantified in serum using ELISA kits that are available commercially (Abcam, United States). Using an iMark Microplate Reader (BIORAD, United States), serum levels of reduced glutathione and superoxide dismutase, two indicators of oxidative stress, were examined. The Supplementary material describes certain procedures in detail.

### Estimation of sample size

Patients were recruited to this pilot study from the movement disorders clinic for a total of 36 months. As a pilot study and without prior knowledge of the predicted treatment impact (and variability) of a month of nVNS a formal power calculation was not considered necessary.

### Security and adherence

Through the reporting of adverse events and subsequent causality analyses using set WHO-UMC standards, patient safety was evaluated. At each appointment, sitting blood pressure and pulse were recorded for each patient. The patients were instructed to fill out a paper diary to note negative incidents.

### Statistical analysis

For parametric data, the mean (and standard deviation) and for nonparametric data, the median (and interquartile range) were used to present clinical and demographic information. The Shapiro–Wilk test (as well as distribution histograms) were used to determine whether the data were normal. Percentages were used to depict categorical data. Left and right gait characteristics were pooled and averaged if there was no side-to-side difference. Using the Wilcoxon Sign Rank test, differential carryover effects between the two sequences were investigated. Because each intervention in the study was only for 1 month, period effects were not anticipated (Karl et al., 2020). The percentage change of the outcome variables from each period was combined, regardless of the order in which the devices were allocated. The Wilcoxon signed rank test was used to assess changes in absolute values of outcome measures (such as biomarkers and clinical rating scores) following the application of active or sham nVNS. The Wilcoxon signed rank test for paired samples was used to examine the percentage change in outcomes from baseline between the active nVNS and sham groups. Fisher’s Exact Test was used to compare categorical variables. The threshold for statistical significance was defined as a *p* value of 0.05. The Benjamini Hochberg correction for multiple comparison method was used (Benjamini and Hochberg, 1995). Statistical analysis was completed using SPSS version 20 (IBM, United States).

## Results

Thirty six participants were enrolled in this cross-over trial; 17 were initially randomized to receive active nVNS and 19 received sham nVNS. Three patients withdrew from the study after the initial screening and randomization procedures. Twenty-one patients successfully completed both arms of the cross-over trial and had thus received both active and sham nVNS by the end of the study (Figure 1 – consort diagram). All participants who finished one or both periods were included in the pre-post analysis. At the conclusion of the study, there were twenty-five pairs of pre-post data for sham nVNS and twenty-one pairs for active nVNS. The 21 patients who finished both arms of the cross-over study were also subjected to an inter-group comparison of the primary outcome measures.

Between sham nVNS and active nVNS, the mean UPDRS III score did not differ at baseline (40.3 vs. 38.5, *p* = 0.328). Table 1 displays the baseline summary scores contrasting the two groups. Table 1 also includes information on demographics, gait measures, clinical traits, and serum marker levels, none of which at baseline differed significantly across groups. Table 2 compares the differences between individual outcome measures (gait parameters and clinical features) for the two groups before and after intervention (active and sham nVNS).

**Table 1:** Comparing the baseline characteristics of demographics, clinical characteristics and serum biomarkers between active and sham nVNS groups.

	Both groups Mean (SD)	Baseline – Sham Group Mean (SD)	Baseline – Active Group Mean (SD)	Group Comparisons (*p* value)
Demography				
Age (years)	62.5 ± 10.3	60.8 ± 14.4	62.26 ± 10.5	1.0
Sex (n) (female)	3 (10.2%)	3 (11.5%)	2 (8.7%)	1.0
Gait				
Velocity (cm/s)	64.5 ± 20.6	66.9 ± 19.4	61.9 ± 20.3	0.13
Average Step Length (cm)	25 ± 20.5	36.8 ± 10.4	36.2 ± 10.3	0.3
Average Stance time (s)	0.8 ± 0.2	0.8 ± 0.2	0.8 ± 0.2	0.03
Clinical scores				
MDS-UPDRS I	15.9 ± 7.3	15.6 ± 6.8	16.3 ± 8.1	0.67
MDS-UPDRS II	21.4 ± 5.5	20.8 ± 5.8	22.1 ± 5.1	0.14
MDS-UPDRS III	39.5 ± 11.6	40.3 ± 12.7	38.5 ± 10.4	0.33
H & Y	2.4 ± 0.6	2.4 ± 0.5	2.3 ± 0.7	0.26
TUG (s)	42 ± 55.2	39.2 ± 77.5	45.4 ± 67.5	0.71
FES	55.2 ± 10.6	54.2 ± 12.8	56.4 ± 7.3	0.25
MMSE	26.4 ± 3.9	25.9 ± 3.8	26.5 ± 3.8	0.92
RBDSQ	4.7 ± 2.9	4.9 ± 2.9	5.2 ± 2.9	0.27
FOGQ1	2.9 ± 0.5	2.9 ± 0.53	2.9 ± 0.54	1.00
FOGQ2	2.7 ± 0.5	2.6 ± 0.5	2.8 ± 0.5	0.16
FOGQ3	3.2 ± 0.7	3.1 ± 0.6	2.5 ± 0.7	0.26
FOGQ4	2.5 ± 0.9	2.4 ± 0.8	2.6 ± 0.9	0.61
FOGQ5	2.3 ± 0.9	2.3 ± 0.8	2.4 ± 1.1	0.88
FOGQ6	2.4 ± 0.9	2.3 ± 0.8	2.5 ± 0.9	0.45
Total FOG-Q score	14.7 ± 5.4	15.5 ± 3.1	13.9 ± 6.9	0.38
Biomarkers				
Serum TNF-α (pg/ml)	25.6 ± 4.1	23.2 ± 2.2	28.1 ± 4.1	0.1
Serum reduced glutathione (pg/ml)	6.4 ± 0.7	6.7 ± 0.7	6.0 ± 0.6	0.3
Serum BDNF (pg/ml)	1945.2 ± 256.6	1943.7 ± 348.1	1943.7 ± 146.4	0.3

The differences were assessed by Wilcoxon Sign Rank Test for numerical variables and Fisher’s exact test for categorical variables (e.g., sex); *p* < 0.05 (*) was considered significant. [SD, Standard Deviation; MDS-UPDRS, MDS-Unified Parkinson’s disease Rating Scale; H&Y, Hoehn and Yahr scale; TUG, Timed Up and Go test; FES, Falls Efficacy Scale; MMSE, Mini Mental State Examination; RBD-Q, REM Sleep Behavior Disorder Questionnaire; FOG-Q, Freezing of Gait Questionnaire; TNF-α, Tumor Necrosis Factor-α; BDNF, Brain Derived Neurotrophic Factor].

**Table 2:** Pre-post differences in clinical profile and gait characteristics for active nVNS and sham nVNS groups.

Clinical outcome variables	Baseline (Pre for nVNS) Mean (SD)	Post-intervention nVNS Mean (SD)	*p* value pre-post nVNS	Baseline (Pre for sham) Mean (SD)	Post-intervention sham Mean (SD)	*p* value pre-post sham
Gait outcome variables						
Velocity	61.9 ± 20.3	72 ± 19.1	0.003*	66.6 ± 20.3	68.31 ± 18.2	0.689
Step length	36.2± 10.3	40.3±10.15	0.007*	36.8 ± 10.4	37.2 ± 10	0.797
Swing time variability	0.04±0.02	0.03 ± 0.02	0.085	0.04 ± 0.03	0.04 ± 0.03	0.432
Step time	0.6±0.10	0.57 ± 0.08	0.003*	0.57 ± 0.099	0.55 ± 0.08	0.059
Swing time	0.37±0.06	0.38 ± 0.07	0.970	0.36 ± 0.07	0.35 ± 0.07	0.338
Stance time	0.83±0.17	0.75 ± 0.12	0.001*	0.77 ± 0.16	0.74 ± 0.12	0.304
Stride velocity variability	6.4±3.2	6.9 ± 3.4	0.846	6.9 ± 2.55	6.9±2.43	0.841
Step length variability	3.9±1.5	4. ± 2.3	0.440	4.1 ± 1.2	4.3 ± 1.3	0.543
Step time variability	0.05 ± 0.03	0.04 ± 0.02	0.114	0.05 ± 0.03	0.05 ± 0.035	0.920
Step time asymmetry	0.04 ± 0.04	0.02 ± 0.02	0.056	0.03 ± 0.03	0.03 ± 0.04	0.819
Step length asymmetry	3.1 ± 2.4	2.5 ± 2.2	0.149	2.7 ± 2.4	2.7 ± 2.1	0.808
Step width	11± 2.9	10.7± 2.9	0.357	10.8 ± 2	10.7 ± 3.7	0.424
Clinical characteristics						
MDS-UPDRS I	16 ± 8	13 ± 7	0.004*	16 ± 7	13 ± 8	0.030
MDS-UPDRS II	22 ± 5	18± 5	0.001*	21 ± 6	17 ± 7	0.009*
MDS-UPDRS III	39 ± 10	32 ± 12	0.002*	40 ± 1	33 ± 1	0.002*
H & Y	2 ± 0.7	2 ± 0.7	0.083	2 ± 0.5	2 ± 0.5	0.655
TUG (s)	45± 67	35± 47	0.033	39 ± 77	42 ± 101	0.098
FES	56 ± 7	50± 8	0.001*	54 ± 13	48 ± 13	0.003*
MMSE	26 ± 4	27 ± 3	0.195	26 ± 4	25 ± 6	0.905
RBDSQ	5.2 ± 2.9	4.1 ± 3	0.036	4 ± 2.8	3.6 ± 2.9	0.177
Total FOG-Q score	16.5 ± 3.5	13.2 ± 3.9	0.001*	15.5 ± 3	11.9 ± 4.3	0.001*
DRS Total	124.8 ± 14.8	120.6 ± 28.9	0.727	120 ± 18.4	114 ± 31.6	0.819

The differences were assessed by Wilcoxon Sign Rank Test; *p* < 0.05 (*) was considered significant after correction for multiple comparisons [SD, Standard Deviation; MDS-UPDRS, MDS-Unified Parkinson’s disease Rating Scale; H&Y, Hoehn and Yahr scale; TUG, Timed Up and Go test; FES, Falls Efficacy Scale; MMSE, Mini Mental State Examination; RBDSQ, REM Sleep Behavior Disorder Screening Questionnaire; FOG-Q, Freezing of Gait Questionnaire].

According to a pairwise pre-post analysis, velocity increased by 16% (*p* = 0.018), step length increased by 11% (*p* = 0.021) and step time decreased by 16% (*p* = 0.003) in the active nVNS group, whereas changes in velocity (2.3%, *p* = 1.0), step length and step time (1.7%, *p* = 0.708) were not significant for the sham nVNS group. With active nVNS but not sham nVNS, velocity (*p* = 0.018), step time (*p* = 0.012), and step length (*p* = 0.021) all significantly improved.

Clinical outcome measures improved considerably in both groups when we evaluated the change in clinical scores before and after therapy in the two groups independently. Both groups showed a significant improvement in the UPDRS II, III, the falls efficacy scale score, and the FOGQ score.

Less than one-third of individuals with FOG experienced freezing episodes while having their gait evaluated (recorded on camera simultaneously). The average length of freezing episodes when walking around the laboratory gait assessment circuit (see Supplementary Figure S1A) decreased from 21 ± 47 to 15 s ± 37 s in the active nVNS group (*p* = 0.042) but did not change significantly after the sham nVNS intervention (27 ± 67 to 72 ± 268 s; *p* = 0.575). However, neither group had a clinically significant change as a result of the average difference in freezing time. The overall amount of time needed to complete the laboratory gait assessment circuit did not differ substantially between the sham nVNS group (128 ± 130 to 159 ± 299 s; *p* = 0.968) and the active nVNS group (116 ± 55 to 94 ± 32 s; *p* = 0.007). The baseline average times for the active nVNS and sham nVNS groups to complete the laboratory gait assessment circuit were 130 and 116 s; (*p* = 0.897), respectively.

**Figure 2:**
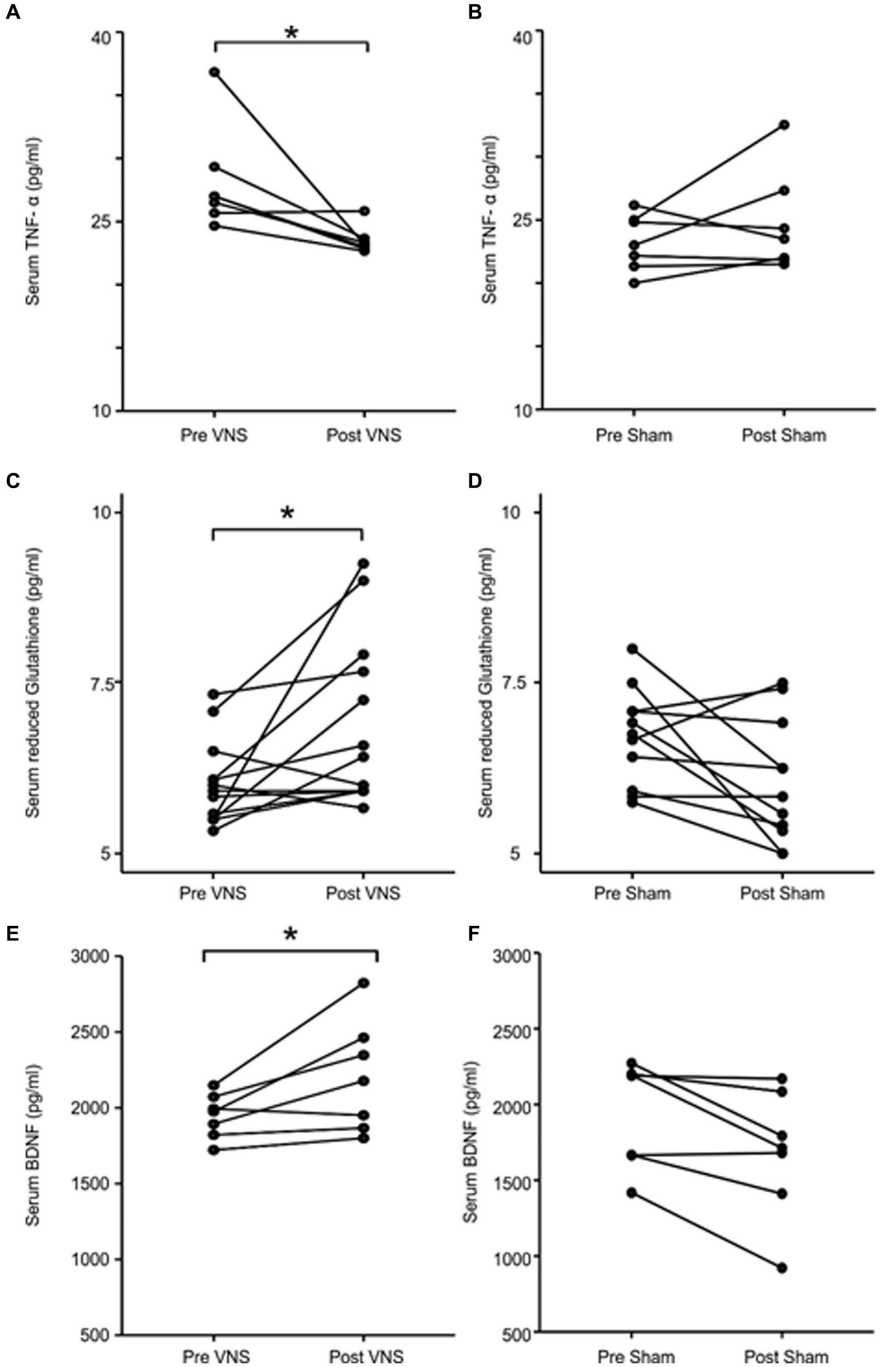
Comparing levels of serum biomarkers before and after intervention in the active and sham nVNS groups. **(A,C,E)** The change in serum TNF-*α*, reduced glutathione and BDNF concentration after active nVNS compared to baseline. **(B,D,F)** The change in serum TNF-*α*, reduced glutathione and BDNF concentration after sham nVNS compared to baseline. Statistical differences were assessed using the Wilcoxon Sign Rank Test, where *p* < 0.05 (*) was considered significant.

Among the biochemical parameters, TNF-α levels were significantly decreased from baseline in patients receiving active nVNS (28.1 to 23.5 pg./mL; *p* = 0.028) but not in those receiving the sham nVNS intervention (23.2 to 24.7 pg./mL; *p* = 0.499; Figures 2A,B). As demonstrated in Figures 2C,D, the reduced glutathione concentration rose following active nVNS (6.1 to 6.8 pg./mL, p = 0.02) but remained relatively unchanged following sham nVNS stimulation (6.7 to 6.1 pg./mL, *p* = 0.05). The active nVNS intervention significantly raised BDNF levels (1946.7 to 2204.1 pg./mL, *p* = 0.028), but decreased with sham nVNS stimulation (1943.7 to 1682.7 pg./mL, *p* = 0.028) as demonstrated in Figures 2E,F. Between groups, there were no appreciable variations in the concentrations of IL-6 (*p* = 0.128), IL-10 (*p* = 0.108), or the specific activity of superoxide dismutase (*p* = 0.058).

**Figure 3:**
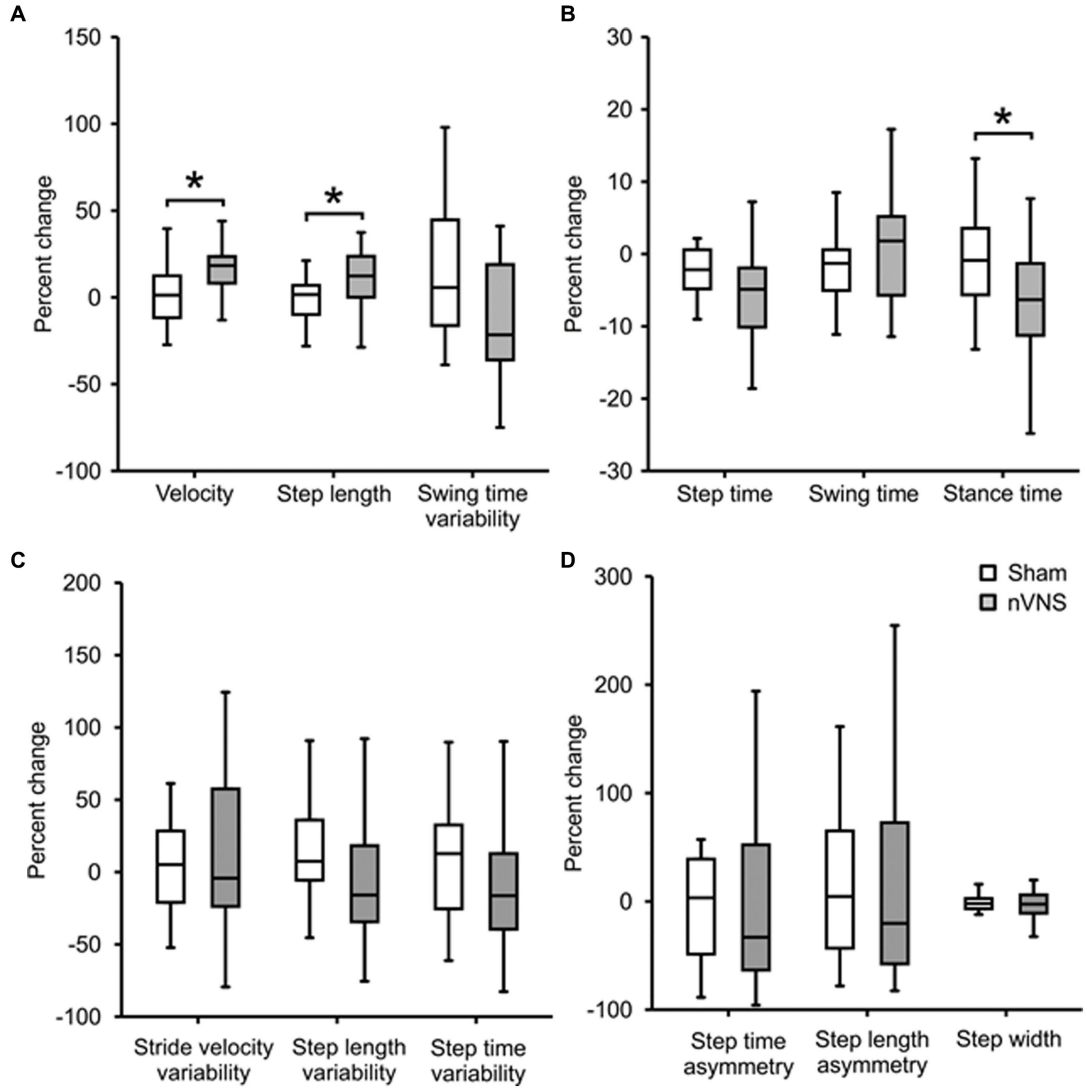
Comparing the percentage change in gait parameters between active and sham nVNS groups. Representative gait parameters are presented. **(A)** Percentage change (from baseline) in gait parameters from the ‘pace’ domain between the active nVNS and sham nVNS groups. **(B)** Percentage change (from baseline) in gait parameters from the “rhythm” domain for active and sham nVNS groups. **(C)** Percentage change (from baseline) in gait parameters from the ‘variability’ domain. **(D)** Percentage change (from baseline) in gait parameters from the ‘asymmetry’ and ‘postural control’ domains. Differences were assessed statistically using the Wilcoxon Sign Rank Test, where *p* < 0.05 (*) was considered significant.

Figure 3 displays percentage changes in gait parameters relative to the starting point. Between the active and sham nVNS groups, we discovered significant changes in step length (*p* = 0.017), stance duration (*p* = 0.006) and the percentage change in velocity (*p* = 0.014).

**Figure 4:**
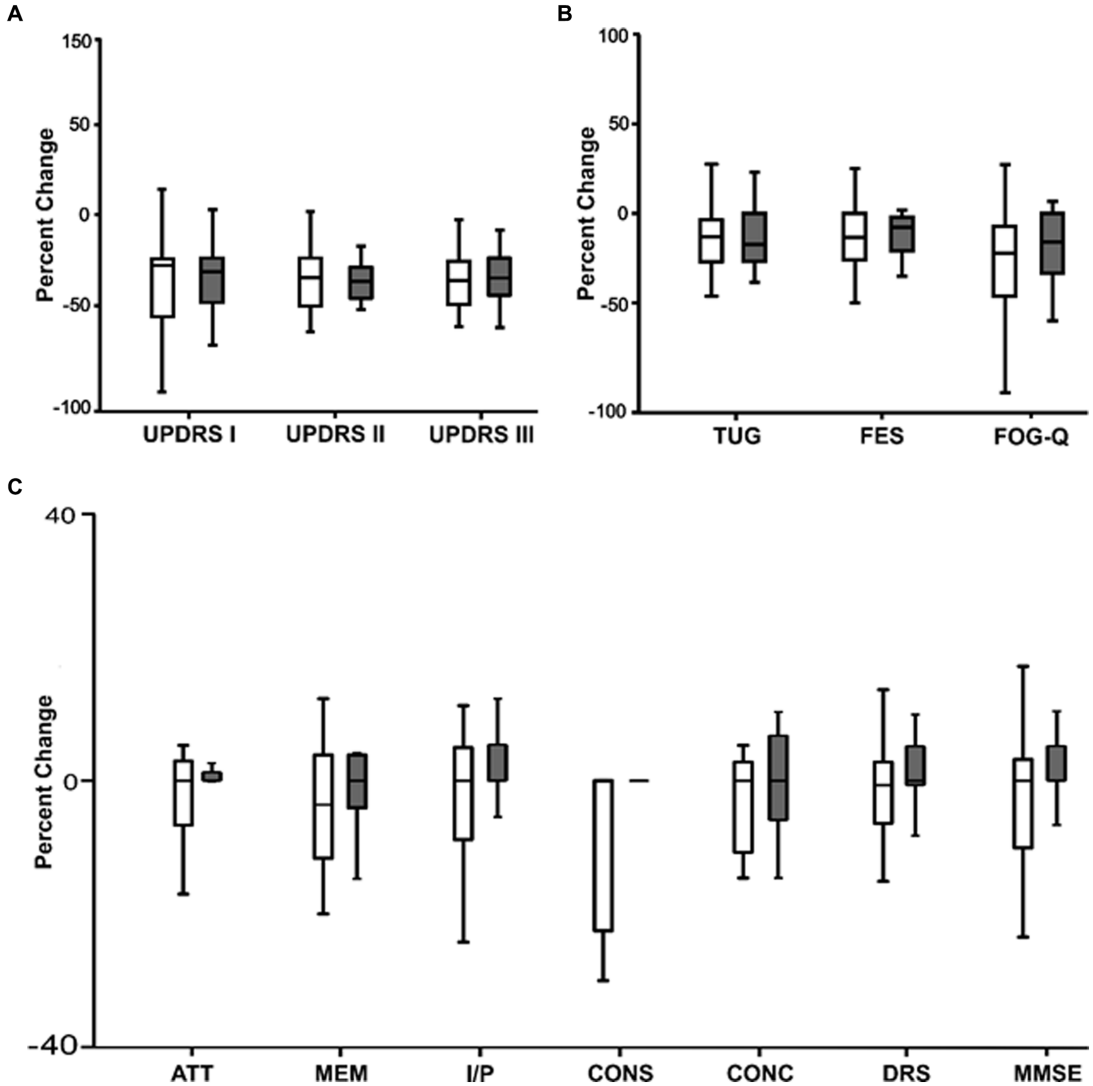
Comparing the percentage change (from baseline) in clinical characteristics between active and sham nVNS groups. **(A)** Percentage change (from baseline) in MDS – Unified Parkinson’s disease Rating Scale (UPDRS Part I, II, III) between active and sham nVNS groups. **(B)** Percentage change (from baseline in time taken for Timed Up and Go Test TUG, Falls Efficacy Scale) (FES score, and Freezing of Gait Questionnaire (FOG-Q) score between active and sham nVNS groups. **(C)** Percentage change (from baseline) in total Dementia Rating Scale (DRS) score and scores in specific domains (ATT, MEM, I/P, CONS, CONC) and Mini Mental State Examination (MMSE) score between active and sham nVNS groups [AAT, Attention; MEM, Memory; I/P, Initiation and Perseveration; CONS, Construction; CONC, Conceptualisation]. Statistical differences were assessed using the Wilcoxon Sign Rank Test, where *p* < 0.05 (*) was considered significant.

In Figure 4 we compared the percentage change in clinical scores between the active and sham nVNS treatments. The percentage change in the clinical ratings did not significantly differ across the groups.

Unexpected results emerged from patient perceptions of their experiences with freezing and their fear of falling as measured by the FOGQ and the falls efficacy scales, respectively. The six gait-freezing questionnaire items and the mean score significantly decreased in both groups. In the sham nVNS and active nVNS groups, the overall FOGQ scores decreased by 26.3% (*p* = 0.001) and 21% (*p* = 0.001), respectively. Following active and sham nVNS the mean falls efficacy scale scores decreased by 10.7% (*p* = 0.001) and 12% (*p* = 0.003) respectively.

Between the two groups, there was a comparable percentage change in cognitive scores (Figure 4C). For each group independently calculated, the difference between the raw scores before and after the treatment was not statistically significant.

With either intervention, there was no carry over effect (Supplementary Tables S1, S2).

## Discussion

**Figure 5:**
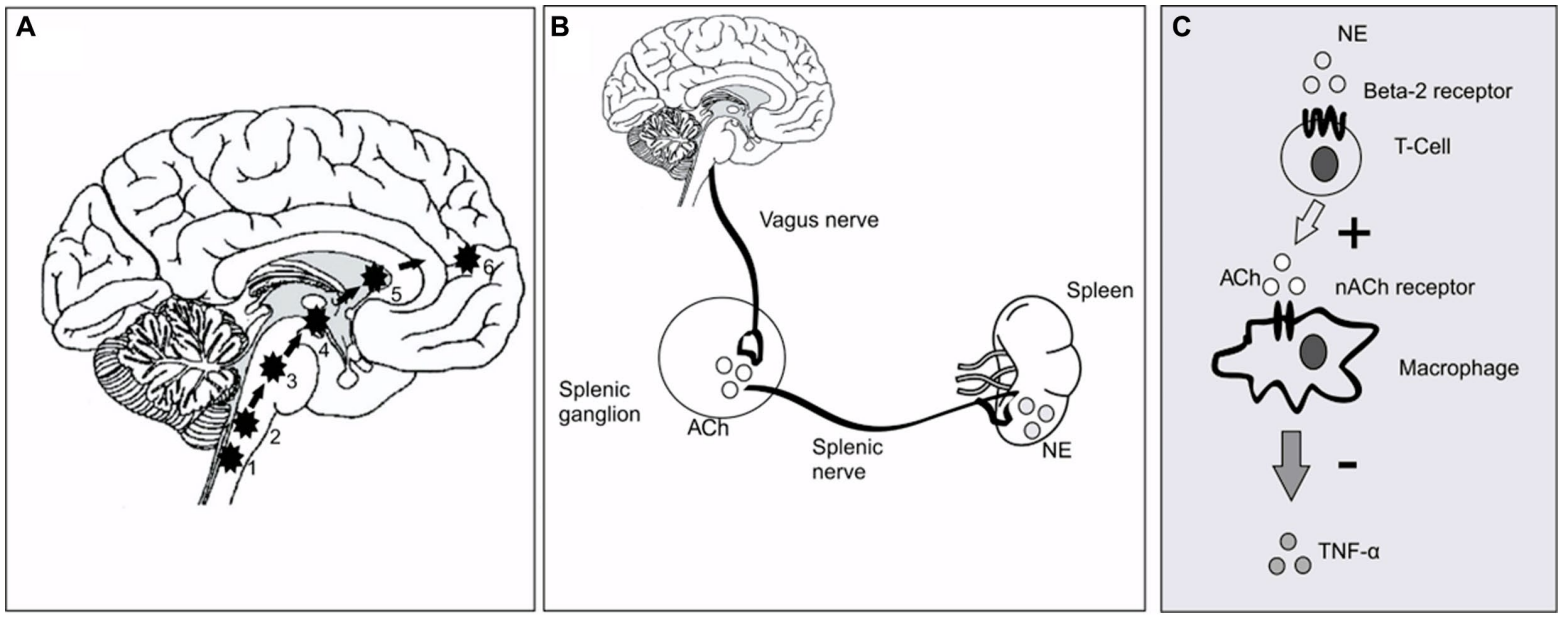
Putative mechanism of nVNS action at circuit level and cellular level. **(A)** The pathway of direct stimulation of brain regions. 1&2, Dorsal motor nucleus of the vagus and *nucleus tractus solitarius*; 3, *Locus coeruleus*; 4&5, Basal ganglia and thalamus; 6, forebrain cholinergic nucleus (including *nucleus basalis* of Meynert). **(B)** Inflammatory reflex through vagus nerve showing the efferent limb. Vagus nerve stimulation leads to secretion of ACh in the splenic ganglion. ACh in turn stimulates the splenic nerve, which provides direct adrenergic innervation to the spleen [Ach, Acetyl Choline; NE, Norepinephrine/Noradrenaline]. **(C)**. The cellular and molecular environment inside the spleen. NE secreted by splenic nerve stimulates T cells (cholinesterase positive to secrete Ach). The secreted neurotransmitter binds with the 7-α subunit of nicotinic ACh receptors on the surface of macrophages and inhibits secretion of TNF-*α*.

This is the first randomized, double-blind, sham-controlled study to attest to the efficacy of cervical nVNS as an adjunctive treatment for PD. After receiving active nVNS treatment for a month, there were noticeable improvements in gait. The central neuronal networks controlling gait are modulated immediately by nVNS (Figure 5A) but less obvious are the mechanisms by which the long-term effects of nVNS emerge. While the rise in serum BDNF would seem to suggest that neuroplasticity plays a role, the ability of nVNS to reduce pro-inflammatory cytokines such as TNF-α hints at an anti-inflammatory action. The changes in antioxidant levels may also point to disease-modifying effects.

As shown in Figure 5, previous investigations in animals have demonstrated that VNS largely exerts its effects through afferent inputs to the *nucleus tractus solitarius* and subsequent sequential activation of the *locus coeruleus* (Engineer et al., 2011). A noradrenergic nucleus, the *locus coeruleus* projects broadly to cortical and subcortical regions (Frangos and Komisaruk, 2017). If there is direct brain activation through excitatory neurotransmitters such as noradrenaline (Grimbergen et al., 2009), improvements in postural instability and gait in PD would be anticipated. Since the *locus coeruleus* receives afferent input from the forebrain cholinergic *nucleus basalis* of Meynert, which projects cholinergic fibers widely throughout the cerebral cortex, hence cortical cholinergic tone is also likely to be enhanced by nVNS (Engineer et al., 2011). It is interesting to note that deficits of walking speed in PD patients have been linked to diminished cortical cholinergic tone (Rochester et al., 2012). In this study, a walkway with built-in pressure sensors was used to measure the parameters of two-dimensional gait in detail. Based on principal component analysis of gait data from PD patients, gait parameters are often divided into five categories (pace, rhythm, asymmetry, variability, and postural control) (Lord et al., 2014). With nVNS therapy, we saw significant gains in velocity and step length (in the pace domain) and a decrease in stance time (in the rhythm domain), showing that PD patients were walking more quickly and more rhythmically. Other gait metrics significantly improved from baseline, specifically after active nVNS therapy, in all five gait domains, indicating that nVNS improves gait quality across the board for PD patients. The timed up and go test, another quantitative surrogate measure of gait speed, also showed considerable improvement.

Mixed results were obtained from the video-based assessment of gait freezing, one of the key outcome metrics. Although only the active nVNS group experienced a significant decrease in the average length of freezing episodes while moving around the gait assessment circuit in the lab, both groups experienced a significant decline from baseline in the patients’ perceptions of the disability brought on by FOGQ and fear of falling. Therefore, the clinical significance of the changes in freezing duration is unclear. Given the methodological limitations of video-based assessment of gait freezing, this clinically marginal outcome is not wholly surprising. Less than one-third of our patients experienced freezing episodes during video recording, as the severity of freezing can alter over the course of a single clinic visit (Nieuwboer and Giladi, 2008). Additionally, we avoided using methods that would cause FOGQ while we were filming. Gait freezing should ideally be measured over a longer examination time, with covert video capture. This might be done with a wearable monitoring device or by examining extensive domiciliary video records. Such methods might be used in nVNS interventional trials in the future.

We evaluated two crucial non-motor characteristics, cognition and sleep (especially RBD), both of which are worse in PD patients as the disease advances. In order to maintain healthy cognition, basal forebrain cholinergic neurons are critical for controlling attention (Sarter and Bruno, 2004). Additionally, medications that improve cholinergic transmission are frequently used to treat cognitive impairment (Ellis, 2005). One could have anticipated an increase in cognitive performance in the nVNS group as the putative mechanism the putative mechanism is the cholinergic effects of nVNS via *nucleus basalis* of Meynert (Johnson and Wilson, 2018). While there have been conflicting findings on how VNS affects cognition (Rizzo et al., 2003), the majority of studies have failed to show any appreciable improvements in cognition in patients receiving VNS as a supplementary therapy for epilepsy (Dodrill and Morris, 2001). The main drawback of such research is the short follow-up period; with less than a year of continuous treatment, it is challenging to detect meaningful cognitive gain (or a slower rate of deterioration/progression). Given the relatively brief duration of nVNS treatment, the lack of improvement in cognitive tests in our group of patients is therefore not wholly unexpected. With nVNS, RBD might likewise be anticipated to improve, especially in light of findings pointing to the *locus coeruleus* as a significant anatomical substrate of RBD (García-Lorenzo et al., 2013). Even though we discovered no impacts of nVNS in our study, future research using polysomnography may want to revisit the effects of nVNS on RBD.

Evidence also points to a reflex mechanism (Figures 5B,C) (Tracey, 2009) through which vagal afferent stimulation activates vagal efferent fibers, which in turn trigger splenic T-cells to produce acetylcholine. Consequently, less cytokine is secreted as a result of ACh binding to nicotinic receptors (7-subunit) on the surface of macrophages in and around the spleen. Therefore, as part of this crossover study, we also examined a number of molecular biomarkers of inflammation and redox dysregulation, which have been shown to be upregulated in the serum and cerebrospinal fluid of PD patients (Müller et al., 1998) and to correlate in some studies with the degree of motor dysfunction and the degree of neurodegeneration in PD, raising the possibility that PD is an inflammatory disease (Adams et al., 2019). Despite the fact that we did not track the impact of nVNS on circulating T-cell subsets, we were able to demonstrate that it markedly decreased TNF-α levels and elevated reduced glutathione concentrations. Superoxide dismutase activity and IL-6 and IL-10 levels did not show any appreciable alterations. This might be connected to the stimulation settings (Tsaava et al., 2020). These could be further optimized to have an improved anti-inflammatory impact.

As a peripheral biomarker of neuroplasticity in numerous neurodegenerative illnesses, including PD, BDNF has received immense attention in research. PD patients have considerably lower serum levels of BDNF than age-matched controls and it has been shown that the concentration is negatively correlated with the severity of the disease (Scalzo et al., 2010). It is therefore interesting to note that BDNF is also closely linked to inflammation, suggesting that it may act as a link between neuroplasticity and inflammation (Calabrese et al., 2014). Peripheral BDNF concentration has been employed as a surrogate measure for interventional effects on neuroplasticity in a variety of neurostimulation investigations (Zhao et al., 2019). Following VNS, BDNF expression was increased in rat brain, indicating a potential neuro-modulatory/neuroprotective impact (Follesa et al., 2007). We assessed peripheral BDNF in a subset of patients from our dataset in order to translate this finding and found that BDNF concentration considerably increased following active nVNS.

Overall, our findings offer the first proof that nVNS decreases key pro-inflammatory cytokines, enhances both BDNF and reduced glutathione levels in PD patients, and that nVNS may even have disease-modifying effects in PD. Along with improvements in motor symptoms in PD patients, additional biomarkers, including BDNF, TNF-α, and reduced glutathione may be useful for optimizing nVNS treatment regimens for PD.

The main goals of this study were to ascertain whether a novel intervention could treat PD symptoms that are in general very challenging to treat and, if successful, to bring a potentially useful therapeutic technology to the clinic. Importantly, the treatment should be secure and simple to use. We therefore monitored adverse events to evaluate the safety of nVNS. Fortunately, neither interventional group reported any clinically significant negative device-related effects. Every patient had their blood pressure and pulse tested at each appointment, and there was no significant variation from baseline for either of these vital signs. The effects of stimulating the right vagus nerve on heart rate are negligible and did not pose an additional risk of adverse cardiac effects, despite the fact that we advised patients to stimulate the left vagus nerve to avoid the theoretical risk of adverse cardiac effects (Yamakawa et al., 2014). According to recent research, therapy can be administered safely on either side (Spuck et al., 2008). With the exception of two patients who needed help from their carer to administer nVNS, most patients were happy with the treatment and could self-administer the therapy at the required frequency. Three patients who reported severe discomfort at the lowest stimulator settings withdrew from the study. Two patients who could not tolerate sham stimulation also withdrew from the study. Other participants who also withdrew from the study did so for reasons that had nothing to do with the research equipment or side effects of the intervention.

Although our results are highly encouraging, there are nevertheless some limitations, not least of which is the fact that after correcting for multiple comparisons, we observed no significant difference between groups. This was predicted because the experiment was intended to serve as a pilot study where findings would inform the power calculation for a subsequent trial. Other limitations will also need to be addressed before embarking upon a larger trial of nVNS in PD. These include the measurement of molecular biomarkers in every trial participant, if possible and using ambulatory monitoring devices to overcome the shortcomings of video-based assessment of FOG (as described above). Finally, practical concerns about the delivery of nVNS in elderly populations may need to be addressed in future generations of the device, regardless of whether a carer is required (see above).

This study has offered preliminary proof that nVNS is safe and effective for treating both motor and non-motor symptoms of PD. Future research on nVNS for PD should first determine how long treatment benefits (and potential neuroprotective effects) persist before noticeable motor symptoms reappear in order to optimize treatment parameters in the future. We hope that our promising results will provoke interest and encourage stakeholders to consider collaborating on a larger, definitive multi-center studies of nVNS in PD.

## Data availability statement

The raw data supporting the conclusions of this article will be made available by the authors, without undue reservation.

## Ethics statement

The studies involving humans were approved by Institute of Neurosciences Kolkata Ethics Committee. The studies were conducted in accordance with the local legislation and institutional requirements. The participants provided their written informed consent to participate in this study.

## Author contributions

BM: Formal analysis, Methodology, Project administration, Writing – original draft. SC: Data curation, Formal analysis, Investigation, Methodology, Project administration, Supervision, Writing – review & editing. RB: Conceptualization, Investigation, Methodology, Supervision, Writing – review & editing. AR: Data curation, Formal analysis, Project administration, Writing – original draft, Writing – review & editing. KC: Data curation, Formal analysis, Methodology, Project administration, Writing – review & editing. PB: Methodology, Project administration, Writing – review & editing. RS: Methodology, Project administration, Writing – review & editing. SH: Methodology, Project administration, Writing – review & editing. SS: Conceptualization, Investigation, Methodology, Supervision, Writing – review & editing. SB: Conceptualization, Formal analysis, Investigation, Methodology, Project administration, Supervision, Writing – review & editing. MB: Conceptualization, Methodology, Project administration, Supervision, Writing – review & editing. HK: Conceptualization, Funding acquisition, Project administration, Writing – review & editing.
