# Supplementary material for: Effects of non-invasive vagus nerve stimulation on clinical symptoms and molecular biomarkers in Parkinson’s disease
Source: Front Aging Neurosci. 2024 Feb 7;15:1331575. doi: 10.3389/fnagi.2023.1331575 (PMC10879328; doi:10.3389/fnagi.2023.1331575)
Supplement: Supplementary file 1 [file Data_Sheet_1.doc]

## Supplementary material

**Measurement of gait parameters:** Patients were instructed to walk at their preferred walking speed in a semi-circular path where a 5-metre-long gait mat (GaitRite) was placed on the straight edge of the semicircle (see Supplementary Figure 1). Patients followed the same path each time, crossing the gait mat at least six times. Each traversal of the gait mat was considered a single walking trial. The gait data was digitized and stored on a computer for offline analysis of individual gait trials. The first traversal (walking trial) was considered a practice trial and excluded from further analysis and only the next four trials included in further analysis. Walking trials interrupted by episodes of freezing of gait were also replaced by a backup walking trial (i.e. additional sixth trial) for the purposes of analysis. Partial foot falls on the gait mat at the beginning and end of a single walking trial were removed from assessments. The entire gait assessment including the gait mat and Timed Up and Go was recorded on video using a handheld high definition video camera.

From the above-mentioned method, spatiotemporal gait characteristics and certain derived gait parameters were determined. The panel of gait parameters were classified into five gait domains (pace, rhythm, variability, asymmetry and posture) and a total of twelve representative gait characteristics within these domains were used for analysis:

- Pace: step velocity; step length; swing time variability
- Rhythm: step time; stance time; swing time
- Variability: step length variability; step time variability; stance time variability
- Asymmetry: step time asymmetry; step length asymmetry
- Postural control: step width

**Timed up and Go test:** TUG is an objective assessment of gait speed, balance and functional level related to locomotion and balance. The test starts with the participant sitting correctly (hips at the back of the seat) in a chair with arm rests. On the word ‘GO’ he/she stands up, walks for three meters to a line on the floor, turns around, walks back to the chair and sits down. The patients is also instructed to walk at a regular pace.

**Video analysis of gait:** The extent of freezing of gait was assessed through *post hoc* manual video assessment by a blinded rater trained in movement disorders and gait assessment of Parkinson’s disease patients. The duration of freezing of gait while initiating walking, walking straight, turning and reaching the destination during the TUG test was estimated separately from the video recordings. The total duration of freezing of gait during circular walking was also recorded.

**Assessment of Freezing using FOGQ:** The subjective feeling of freezing of gait over the preceding one month was estimated using the freezing of gait questionnaire.

The freezing of gait questionnaire is commonly used to assess freezing of gait in epidemiological studies and clinical trials. It is unrelated to falls in patients with Parkinson’s disease. This brief six item questionnaire shows moderate correlation with the activity of daily living (ADL) and motor parts of the UPDRS. Individual items are rated using a five-point Likert scale where 0 indicates normal function and 4 indicates worst performance and then summed to produce a total score.

**Motor and non-motor analysis with MDS UPDRS:** The Movement Disorders Society sponsored revised version of Unified Parkinson’s disease Rating Scale (MDS-UPDRS) is the most widely used clinical scale for assessing overall motor and non-motor function/dysfunction and disease severity in patients with Parkinson’s disease.

The MDS-UPDRS has four parts:

Part I: This portion of the scale assesses the non-motor impact of Parkinson’s disease on the patients’ experiences of daily living.

Part II: motor experiences of daily living.

Part III: This portion of the scale assesses the motor signs of Parkinson’s disease

Part IV: In this section, the rater uses historical and objective information to assess two motor complications, dyskinesias and motor fluctuations that include OFF-state dystonia.

**Assessment of falls using the Falls Efficacy Scale-International (FES-I):** Assessment of falls was performed by applying the FES questionnaire. The Falls Efficacy Scale-International (FES-I) is a short, easy to administer tool that measures the level of concern about falling during social and physical activities inside and outside the home. The level of concern is measured on a four-point Likert scale (1=not at all concerned to 4=very concerned).

**Assessment of cognitive function with MMSE and Mattis DRS:** The Mini-Mental State Exam (MMSE) is a widely used test of cognitive function among the elderly; it includes tests of orientation, attention, memory, language and visual-spatial skills. It is a questionnaire with 11 questions with maximum score of 30. A score of 23 or less is indicative of mild cognitive impairment. Mattis DRS evaluates different domains of cognitive function, namely, Attention, Initiation/Perseveration (I/P, Construction, Conceptualization and Memory). The maximum total possible score is 144 points.

**Assessment of Rapid Eye Movement Sleep Behaviour Disorder using (RBDSQ):** The rapid eye movement sleep behaviour disorders screening questionnaire was used to screen for Rapid eye movement Sleep Behaviour disorder in Parkinson’s disease patients. It is a 10-item patient self-rating questionnaire covering the clinical features of rapid eye movement sleep behaviour disorders.  The maximum total score of the rapid eye movement sleep behaviour disorders screening questionnaire is 13, with a higher score suggesting more features associated with rapid eye movement sleep behaviour disorders. A cut-off score of 5 was reported by the authors as most useful when differentiating patients with idiopathic rapid eye movement sleep behaviour disorders from controls.

**Blood sampling and determination of Protein content:** Peripheral venous blood (5ml) was obtained from Parkinson’s disease patients and controls and collected in ethylenediaminetetraacetic acid (EDTA)-free tubes for serum isolation. Serum samples were stored in aliquots at -20°C.

Protein content of serum samples was determined using the Lowry method (Lowry et al., 1951). Samples were analysed using an iMark Microplate Reader, BIORAD, USA.

**Inflammatory profile**: ELISA kits (Abcam, USA) were used to detect serum levels of IL-6, TNF Alpha and IL-10 (pg/ml) for the validation Cohort. Samples were analysed using an iMark Microplate Reader, BIORAD, USA.

**Brain derived neurotrophic factor (BDNF):** Serum brain-derived neurotrophic factor (BDNF) concentration (pg/ml) for the validation cohort was estimated using an ELISA kit (Abcam, USA). Samples were analysed using an iMark Microplate Reader, BIORAD, USA.

**Determination of reduced glutathione (GSH) content:** Glutathione in its reduced form is one of the most potent antioxidants. Serum samples from the primary cohort were treated with 0.1 ml of 25% trichloroacetic acid (TCA) and the resulting precipitate was pelleted by centrifugation at 3,900 x g for 10 mins. The free endogenous sulfhydryl was assayed in a mixture of 3ml in volume (2 ml) of 0.5 mM DTNB prepared in 0.2 M phosphate buffer, with 1 ml of cell supernatant. The thiol group of GSH reacts with DTNB forming a yellow complex whose absorbance was read at 412 nm to determine GSH content (in nMol/ml) of the sample (Moron et al. 1979).

**Determination of superoxide dismutase (SOD) activity:** Superoxide Dismutase is an antioxidant enzyme that scavenges free radicals of hydrogen peroxide (H2O2). Superoxide Dismutase activity (U/mg of protein was determined by the pyrogallol autoxidation method (Marklund et al., 1974) with a slight modification. Briefly, the serum sample was added to tris-cacodylic acid buffer (62.5 mM), followed by the addition of pyrogallol (4 mM). The autoxidation of pyrogallol was monitored at 420 nm. Specific Activity was estimated by dividing the activity by the protein concentration.

**Supplementary Table 1. Carry-over effect of nVNS on clinical and gait outcomes at baseline of both periods.**

| **Clinical and gait outcomes** | **Sequence1 (nVNS first)** | **Sequence1 (nVNS first)** | **P value** | **Corrected p value** |
| --- | --- | --- | --- | --- |
|  | **Preintervention assessment of period 1** | **Preintervention assessment of period 2** |  |  |
| **Clinical Characteristics** | | | | |
| **UPDRS III** | 44.6+14.8 | 40.8+10.1 | 0.075 | 0.525 |
| **H & Y** | 2.5 + .05 | 2.2+ 0.9 | 0.234 | 0.819 |
| **TUG** | 53.8+ 110.6 | 44.6 + 81.4 | 0.301 | 0.702 |
| **FES** | 52.4+ 17.2 | 59.3 + 4.5 | 0.683 | 1.195 |
| **MMSE** | 26.6 + 3.8 | 26.4 + 4.3 | 0.767 | 1.074 |
| **RBD** | 4.8+ 3.0 | 5.6 + 2.9 | 0.798 | 0.931 |
| **FOGQ** | 16.7+ 3.2 | 16.7+ 2.8 | 0.92 | 0.920 |
| **Gait variables** | | | | |
| **Step length asymmetry** | 3+3 | 3+2 | 0.333 | 0.444 |
| **Stride velocity variability** | 6+2 | 7+3 | 0.155 | 0.232 |
| **Swing time variability** | 0.03 + 0.02 | 0.05+ 0.02 | 0.041 | 0.098 |
| **Step Length Variability** | 4+1 | 4+2 | 0.075 | 0.129 |
| **Step Length** | 38+ 10 | 34+ 11 | 0.021 | 0.084 |
| **Stance Time** | 0.80+0.18 | 0.87+0.17 | 0.021 | 0.063 |
| **Velocity** | 66 + 19 | 56+ 18 | 0.016 | 0.096 |
| **Swing Time** | 0.37+ 0.07 | 0.37+ 0.07 | 0.534 | 0.534 |
| **Step time** | 0.58+ 0.11 | 0.62+ 0.09 | 0.041 | 0.098 |
| **Step time asymmetry** | 0.03+0.03 | 0.05+0.04 | 0.477 | 0.520 |
| **Step Width** | 11+3 | 11+3 | 0.388 | 0.466 |
| **Step Time Variability** | 0.04+0.03 | 0.62+0.09 | 0.002 | 0.024* |

**Supplementary table 2. Carry-**over effect of sham on clinical and gait outcome at baseline of both periods.

| **Clinical and gait outcomes** | **Sequence2 (sham first)** | **Sequence2 (nVNS first)** | **P value** | **Corrected p value** | |
| --- | --- | --- | --- | --- | --- |
|  | **Preintervention assessment of period 1** | **Preintervention assessment of period 2** |
| **Clinical Characteristics** | | | | | |
| **UPDRS III** | 36.7 + 9.7 | 35.4+ 10.6 | 0.116 | 0.812 | |
| **H & Y** | 2.4+ 0.5 | 2.4 + 0.5 | 0.237 | 0.829 | |
| **TUG** | 25.7 + 19.8 | 46.4 + 47.7 | 0.507 | 1.183 | |
| **FES** | 55.9 + 7.1 | 52.4+ 8.7 | 0.611 | 1.069 | |
| **MMSE** | 25.3+ 3.9 | 26.6+3.3 | 0.683 | 0.956 | |
| **RBD** | 3.8+2.7 | 4.7+ 3.0 | 0.713 | 0.831 | |
| **FOGQ** | 14.2+ 2.9 | 11.6+ 9.0 | 1.000 | 1.000 | |
| **Gait variables** | | | | | |
| **Step length asymmetry** | 0.05+ 0.03 | 0.58+ 0.11 | 0.008 | | 0.096 |
| **Stride velocity variability** | 5.0+ 1.0 | 3.0+ 1.0 | 0.015 | | 0.090 |
| **Swing time variability** | 0.35+ 0.07 | 0.38+ 0.06 | 0.021 | | 0.084 |
| **Step Length Variability** | 8.0 + 3.0 | 6.0+3.0 | 0.066 | | 0.198 |
| **Step Length** | 0.05 + 0.04 | 0.03 + 0.01 | 0.214 | | 0.514 |
| **Stance Time** | 0.55 + 0.09 | 0.58+ 0.11 | 0.236 | | 0.472 |
| **Velocity** | 36 + 11.3 | 39 + 9.8 | 0.374 | | 0.641 |
| **Swing Time** | 11 + 3.0 | 11 + 3.0 | 0.374 | | 0.561 |
| **Step time** | 0.75+ 0.14 | 0.78 + 0.17 | 0.515 | | 0.687 |
| **Step time asymmetry** | 3.0+2.0 | 3.0+2.0 | 0.515 | | 0.618 |
| **Step Width** | 0.03 + .04 | 0.03+ 0.04 | 0.575 | | 0.627 |
| **Step Time Variability** | 67+ 22.3 | 70+ 21.9 | 0.678 | | 0.678 |


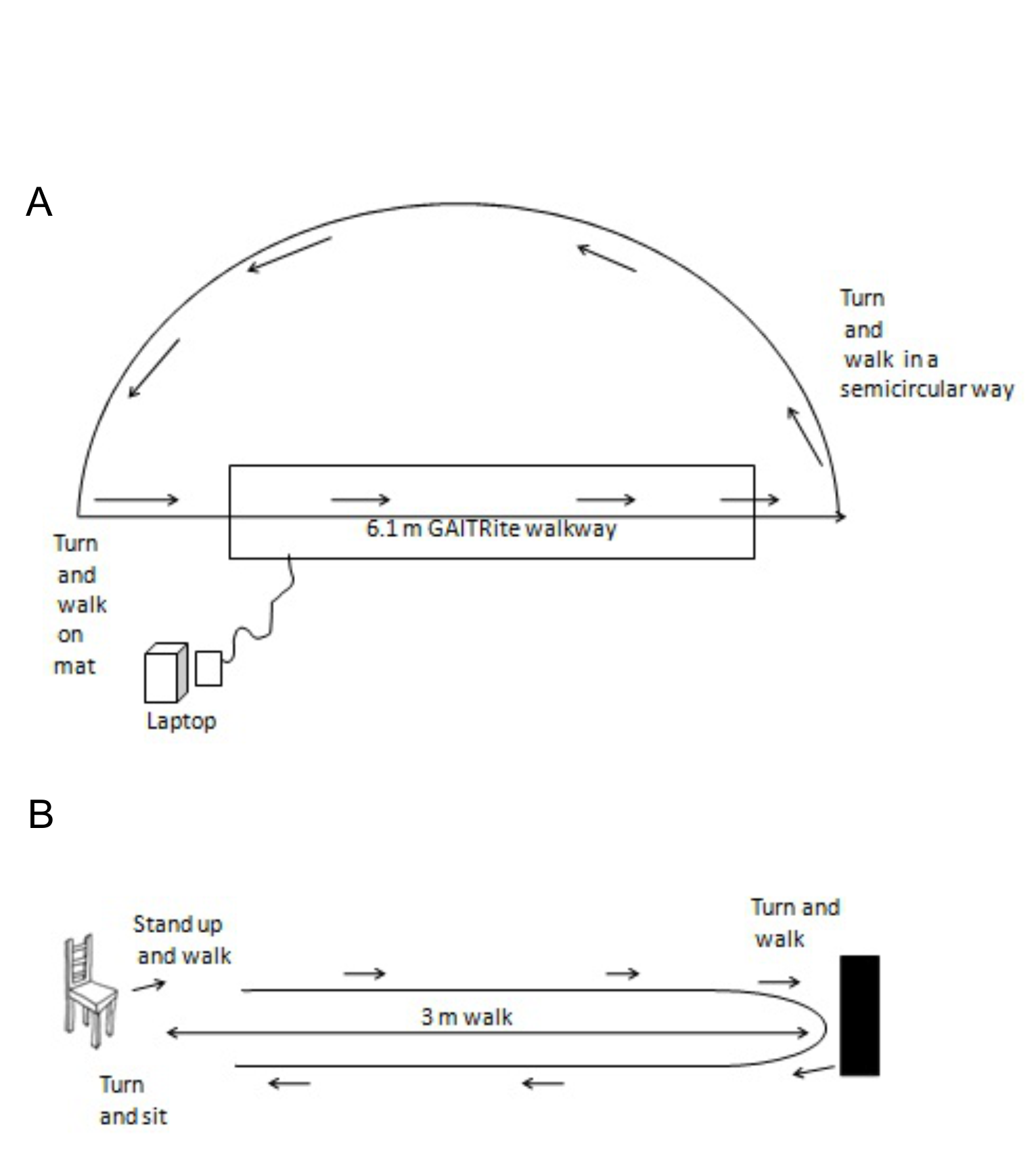


**Supplementary figure 1.Schematic depiction of methods involved in two types of gait analysis.**

**A.** Recording and off-line analysis of two-dimensional gait parameters though electronic gait mat. **B.** Method of Timed Up and Go test.
